# External Validation of the ARCAD Nomogram in a Real-World Cohort with Stage 4 Colorectal Cancer

**DOI:** 10.3390/cancers18183031

**Published:** 2026-09-18

**Authors:** James Yu, Jiannong Li, Michael J. Schell, Pablo Gonzalez Ginestet, John Raymond Zalcberg, R John Simes, Ian Marschner, Richard D. Kim, Katrin Marie Sjoquist

**Affiliations:** 1 Department of Gastrointestinal Medical Oncology, MD Anderson Cancer Center, Houston, TX 77030, USA; jyu6@mdanderson.org; 2 H. Lee Moffitt Cancer Center and Research Institute, Tampa, FL 33612, USArichard.kim@moffitt.org (R.D.K.); 3 NHMRC Clinical Trials Centre, University of Sydney, 119-143 Missenden Rd, Sydney, NSW 2050, Australia; 4 Department of Medical Oncology, Alfred Health and School of Public Health, Faculty of Medicine, Nursing and Health Sciences, Monash University, Melbourne, VIC 3004, Australia; john.zalcberg@monash.edu

**Keywords:** colorectal cancer, ARCAD, nomogram, external validation, prognosis

## Abstract

Introduction: The ARCAD-CRC nomogram predicts 1-year survival in clinical trial populations with stage 4 colorectal cancer (CRC). This study evaluated its performance in a real-world cohort. Methods: Patients with stage 4 CRC were identified from the Flatiron database. A multivariable Cox proportional hazards model for overall survival was applied to fit the reduced model derived from the published full ARCAD nomogram, as some variables were unavailable in the database. Validation of the reduced model used C-index, while predictive performance was assessed using time-dependent ROC analysis and calibration in the Flatiron real-world cohort (FRWC). Results: A total 9710 patients, including 5740 deceased, were analyzed. The FRWC was older (64 vs. 62 years) and had poorer ECOG-PS (15% vs. 4% with ECOG 2+) compared to the ARCAD cohort. The reduced nomogram’s calibration was comparable to the full model (C-index 0.67 vs. 0.68). In the FRWC, the reduced nomogram showed good discrimination, with time-dependent AUROC at the 12-month time horizon of 0.741 (95% CI 0.729–0.753). Calibration plots indicated the reduced ARCAD-CRC nomogram performed quantitively well with a calibration slope of 1.010 (95% CI 0.966–1.054). The calibration intercept was −0.160 (95% CI −0.189, −0.131), indicating systematic underestimation of 1-year survival in the FRWC. The predicted and observed 1-year OS using the reduced ARCAD nomogram in the FRWC were 0.640 (95% CI 0.637, 0.644) and 0.694 (95% CI 0.684–0.703), respectively. Conclusions: Our findings show that the ARCAD-CRC nomogram is a useful tool for predicting 1-year survival in a real-world cohort. The observed underestimation of survival may be due to advances in CRC treatments, as the FRWC cohort (2013–2020) was collected later than the original ARCAD cohort (1997–2012).

## Simple Summary

We sought to evaluate the performance of the ARCAD-CRC nomogram in a real-world cohort. The Flatiron database was used to assess the predictive performance, calibration and discrimination of the nomogram to predict 1-year survival. Our findings indicate that the ARCAD-CRC nomogram is a useful tool for predicting 1-year survival in a real-world cohort. The observed underestimation of survival may be due to recent advances in CRC management, as the FRWC cohort (2013–2020) was collected later than the original ARCAD cohort (1997–2012).

## 1. Introduction

Globally, colorectal cancer (CRC) is the third most prevalent cancer and the second leading cause of cancer-related mortality [1]. Similarly, in the United States, CRC holds the fourth position in cancer prevalence and ranks second in cancer-related deaths [2]. Within the US, 23% of colorectal cancer cases are diagnosed at stage 4, with a reported 5-year survival rate of approximately 16.9% [3]. Despite the widespread use of the TNM staging system for prognosis prediction, patients at the same TNM stage exhibit diverse clinical outcomes, highlighting the impact of additional factors on survival. Previous studies have identified demographic features (e.g., age and gender), clinical factors (e.g., performance status), laboratory factors (e.g., albumin), and genomic factors (e.g., *RAS*, *RAF* mutations) as significant contributors to the variation in survival among patients with colon cancer [4,5,6,7,8].

A nomogram serves as a statistical predictive tool, estimating the likelihood of specific future events, such as survival probability, by integrating multiple contributing factors [9]. Nomograms have been used extensively across various cancer types for prognostication, including CRC [10]. In 2018, the ARCAD group developed a nomogram designed to predict 1-year survival probability in stage 4 CRC, encompassing 17 demographic, clinical, tumor burden, laboratory, and genomic factors [11]. Compared to the conventional TNM staging system, this nomogram incorporates well-established prognostic factors such as age, performance status, albumin levels, and *RAS/RAF* mutation status, which can potentially provide a novel prognostication [4,5,6,7,8]. However, a notable limitation is that the ARCAD nomogram was developed using clinical trial populations, which inherently exhibit distinct characteristics, such as better performance status or fewer comorbidities, compared to the real-world population due to the selection criteria of clinical trials [12,13,14].

External validation of the proposed nomogram is thus crucial to ensure its generalizability. To our knowledge, there has been no peer-reviewed published data on the external validation of the ARCAD CRC nomogram. We conducted external validation on a large real-world cohort of ARCAD CRC nomogram in the US.

## 2. Materials and Methods

### 2.1. Original ARCAD CRC Nomogram

The original ARCAD CRC nomogram used data from 22,674 patients with stage 4 CRC from 26 randomized phase III clinical trials accrued between 1997 and 2012 [11]. A 10% holdout sample of 2257 patients was used for external validation, leaving 20,417 patients for model development, which was the cohort used in the current analysis.

The original ARCAD CRC nomogram was designed to predict one-year survival probability by employing a multivariable Cox proportional hazards model. The nomogram includes seventeen variables: baseline age, sex, body mass index, performance status, colon vs. rectal cancer, prior (adjuvant) chemotherapy, number and location of metastatic sites, tumor mutation status (*BRAF*, *KRAS*), bilirubin, albumin, white blood cell count, hemoglobin, platelets, and absolute neutrophil count [11]. The final ARCAD CRC nomogram for 1-year survival prediction was well calibrated, with a concordance index of 0.68 for 1-year overall survival in internal validation [11]. The external validation set, based on a 10% holdout sample from each trial, demonstrated a high concordance rate of 72.8% for one-year survival [11]. The formula for the original multivariable Cox proportional hazards models is available at https://doi.org/10.1093/jnci/djx253, and a web-based module can be accessed at https://arcadadvanced.org.

### 2.2. External Validation Cohort with Real-World Population

We utilized the Flatiron database, a nationwide collection of de-identified electronic health records, collected between 2013 and 2020, to establish a retrospective Flatiron real-world cohort. Inclusion criteria consisted of patients who met the following criteria: (1) histologically confirmed stage 4 CRC, (2) age exceeding 18 years, (3) received at least one line of chemotherapy for metastatic colorectal cancer, (4) had no prior therapy for metastatic disease before data collection, and (5) had baseline blood tests available (defined as the most recent blood tests conducted before the initiation of the 1st-line therapy for metastatic disease). Exclusion criteria included (1) the co-existence of an active secondary tumor and (2) insufficient baseline clinical information required for application of the reduced ARCAD nomogram. Patients with missing ECOG performance status were excluded, whereas missing values in other model predictors were subsequently addressed using the MissForest random forest-based imputation approach. The study protocol was approved by the Advarra/WCG Institutional Review Board (IRB #00000971, Protocol No. 00047445), which granted a waiver of informed consent on 11 November 2020 due to the use of de-identified data.

### 2.3. Imputation of Missing Data and Construction of a Reduced ARCAD Nomogram

Certain variables had missing data (Table S1). The proportion of missing data per variable in the FRWC varied from 0 to 32.2% and is summarized in Table S2. To prevent the loss of information and selection bias [15], we employed multiple imputation through the R package MissForest (random forest-based (MissForest) imputation). We applied randomForest backend (MissForest) using the variables list in Table S2, including survival time and event status, which has demonstrated lower imputation error and better preservation of downstream predictive performance than other imputation methods [16]. Based on previous studies reporting the mutual exclusiveness of *BRAF* and *KRAS* mutations in CRC [17], we imputed *KRAS* as wild type in *BRAF* mutant patients and vice versa for *BRAF* status in *KRAS* mutant patients.

The Flatiron dataset lacked information on the prior chemotherapy, number and sites of metastasis, which are variables included in the full ARCAD CRC nomogram model. Consequently, a multivariable Cox proportional hazards model for OS was employed to generate the reduced ARCAD CRC nomogram model, derived from the published full ARCAD CRC nomogram [18]. Characteristics of the subset of patients (*n* = 20,417) used to generate the reduced ARCAD model are outlined in Table S1. To assess robustness of the findings, we performed a complete-case sensitivity analysis. Results are highly consistent with the primary analyses based on the imputed dataset, with similar estimates of discrimination and calibration (Supplementary Figure S2).

### 2.4. Statistical Analysis

Patient variables between the original ARCAD clinical trial population and our Flatiron real-world cohort were subjected to comparison through a chi-square test for categorical variables and the Wilcoxon rank sum test for continuous variables. The observed 1-year overall survival estimate for the FRWC was derived using the Kaplan–Meier method to account for censoring. The reverse Kaplan–Meier estimate of follow-up time in the FRWC was calculated to determine the adequacy of follow-up. External validation of the ARCAD CRC nomogram encompassed discrimination and calibration analyses [19]. The concordance index (C-index) was used to evaluate the discrimination ability of both the original full ARCAD and the generated reduced ARCAD nomogram within the ARCAD cohort. Subsequently, the predictive performance and discrimination of the reduced ARCAD model were assessed in the Flatiron real-world cohort using the area under the time-dependent ROC curve (AUROC). Subsequently, model calibration was visually evaluated using a calibration plot, where well-calibrated predictions align with the 45° line [15,19,20]. A two-sided *p*-value of ≤0.05 was deemed statistically significant. All statistical analyses were executed using R version 4.2.3 (R Foundation for Statistical Computing).

## 3. Results

### 3.1. Study Population

A total of 9710 patients, 5740 of whom were deceased, were included in the Flatiron real-world cohort for external validation of the ARCAD CRC nomogram. The median follow-up time, estimated with the reverse Kaplan–Meier method, was 2.63 years (95% CI 2.53–2.70), providing adequate follow-up for stable estimation of 1-year survival outcomes. The observed 1-year overall survival was 0.694 (95% CI 0.684–0.703). At 12 months, 5297 patients remained at risk. The overall rate of missing data across all independent variables and patients was 8.5%, and the distribution of each variable was preserved through random forest-based missForest imputation (Table S1). The twelve baseline characteristics of patients used for the reduced ARCAD CRC nomogram were compared between the Flatiron real-world external validation cohort and the original ARCAD development clinical trial cohort, as presented in Table 1. The Flatiron real-world cohort was older (64 vs. 62), more likely to be female, had higher BMI, differed on some laboratory levels, and had a poorer ECOG-PS (15% with a score of 2+ vs. 4%) compared to the original ARCAD cohort.

### 3.2. Comparison Between Reduced and Full ARCAD CRC Nomograms

**Figure 1:**
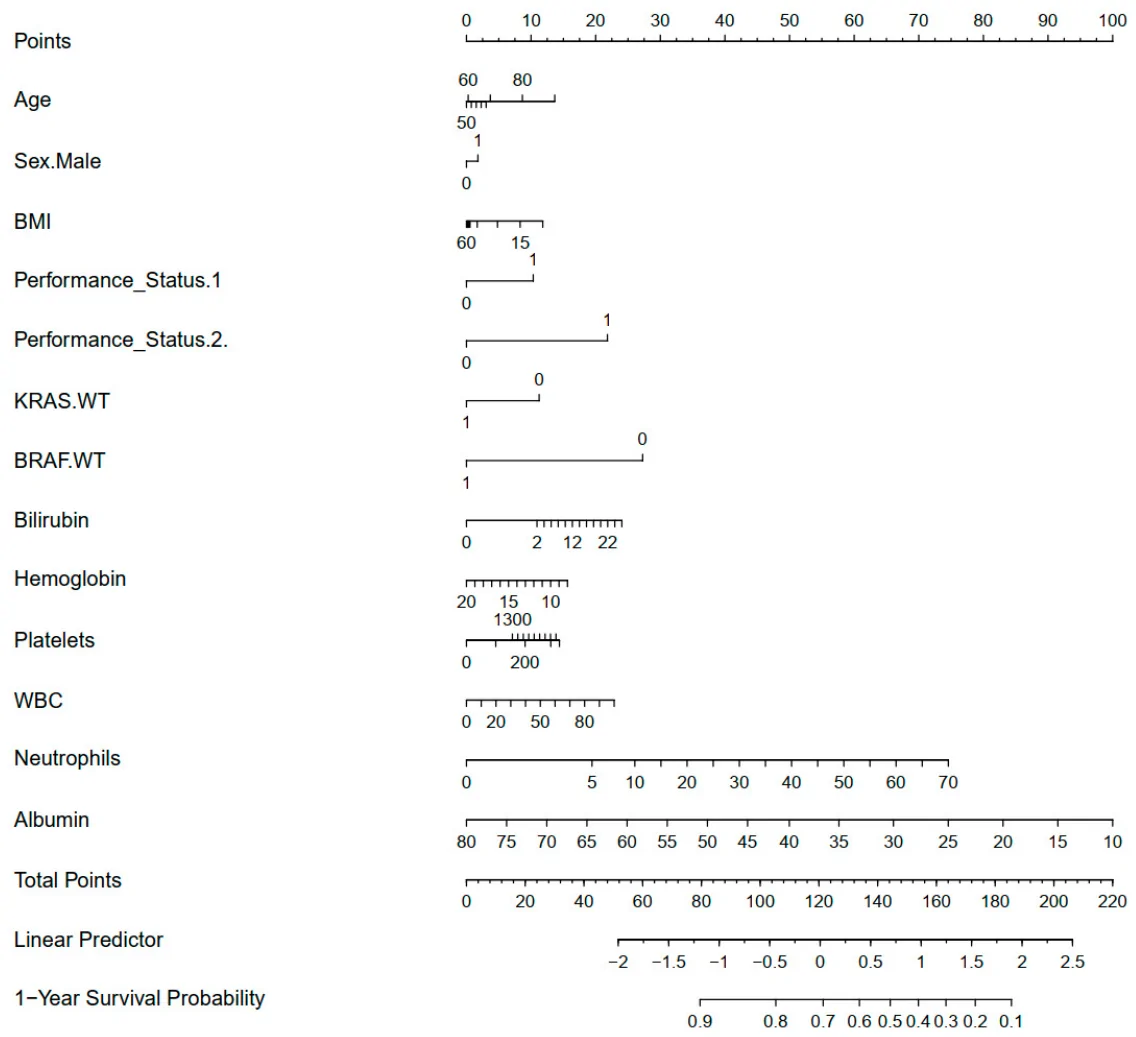
Reduced ARCAD CRC nomogram for 1-year survival prediction.

The reduced ARCAD CRC nomogram for one-year survival prediction in stage 4 CRC was derived from the original full ARCAD CRC nomogram while excluding variables not present in the Flatiron dataset, such as the prior chemotherapy, number of metastatic sites and sites of metastasis (Table 2) [11]. Figure 1 describes the reduced ARCAD CRC nomogram, visualizing the relative prognostic importance of each variable for each outcome [11]. Similar to the previously published full ARCAD CRC nomogram, neutrophils and albumin have the largest impact, while sex has the smallest but still clinically relevant effect on one-year survival prediction [11]. The reduced nomogram showed nearly the same discrimination in the ARCAD population as that of the full nomogram, with C-index values of 0.67 and 0.68, respectively.

### 3.3. Evaluation of the ARCAD CRC Nomogram in a Real-World Population

**Figure 2:**
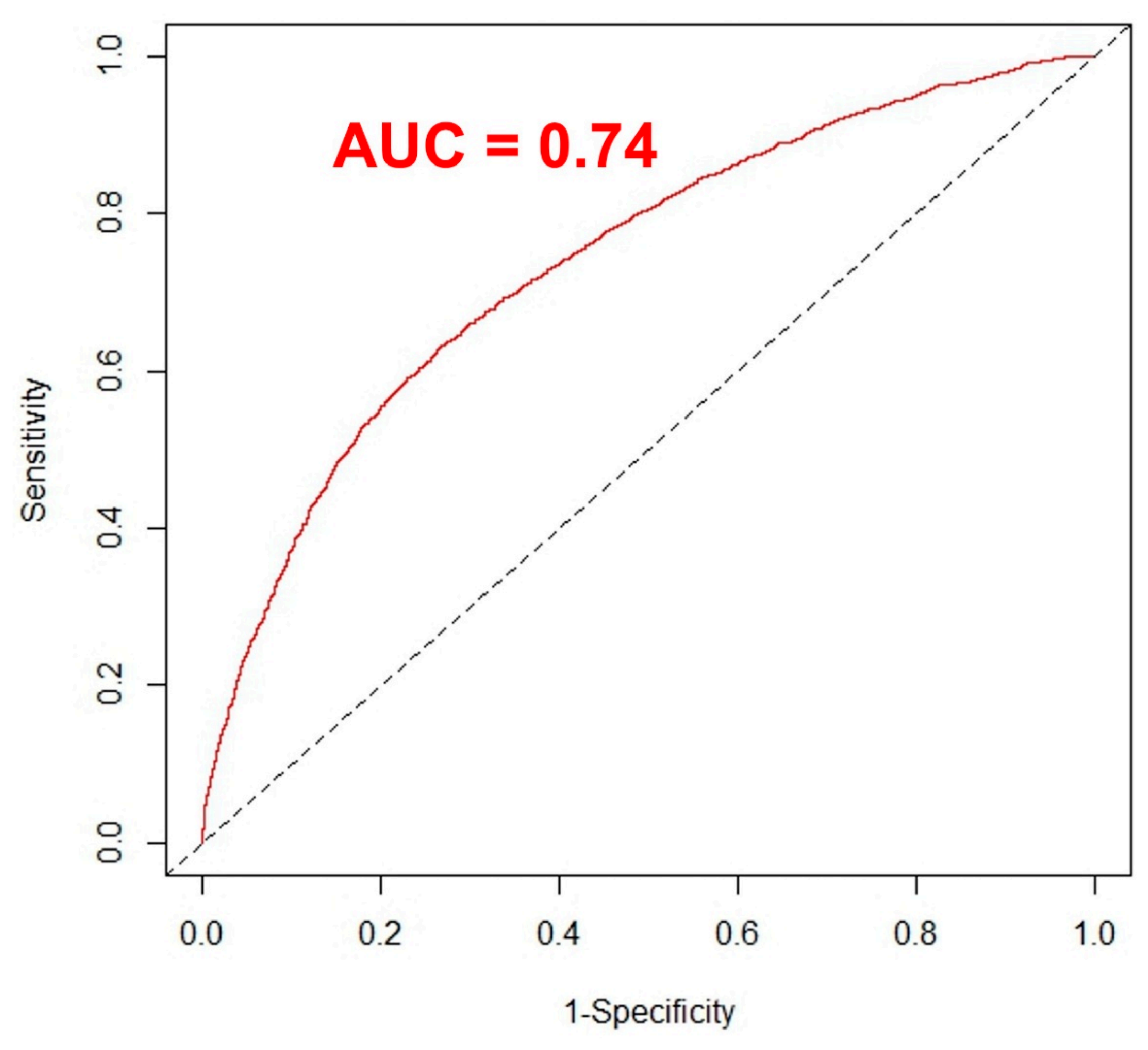
Time-dependent receiver operating characteristic (ROC) curve for the reduced ARCAD CRC nomogram in the Flatiron real-world validation cohort, evaluated at 12 months to assess discrimination. The solid red curve represents the ROC curve of the reduced ARCAD model, with an AUC of 0.741 (95% CI 0.729–0.753). The dashed diagonal line represents the performance expected under random classification (AUC = 0.50). The greater the separation between the ROC curve and the diagonal reference line, the better the model’s ability to discriminate between patients who did and did not experience death within 1 year.

**Figure 3:**
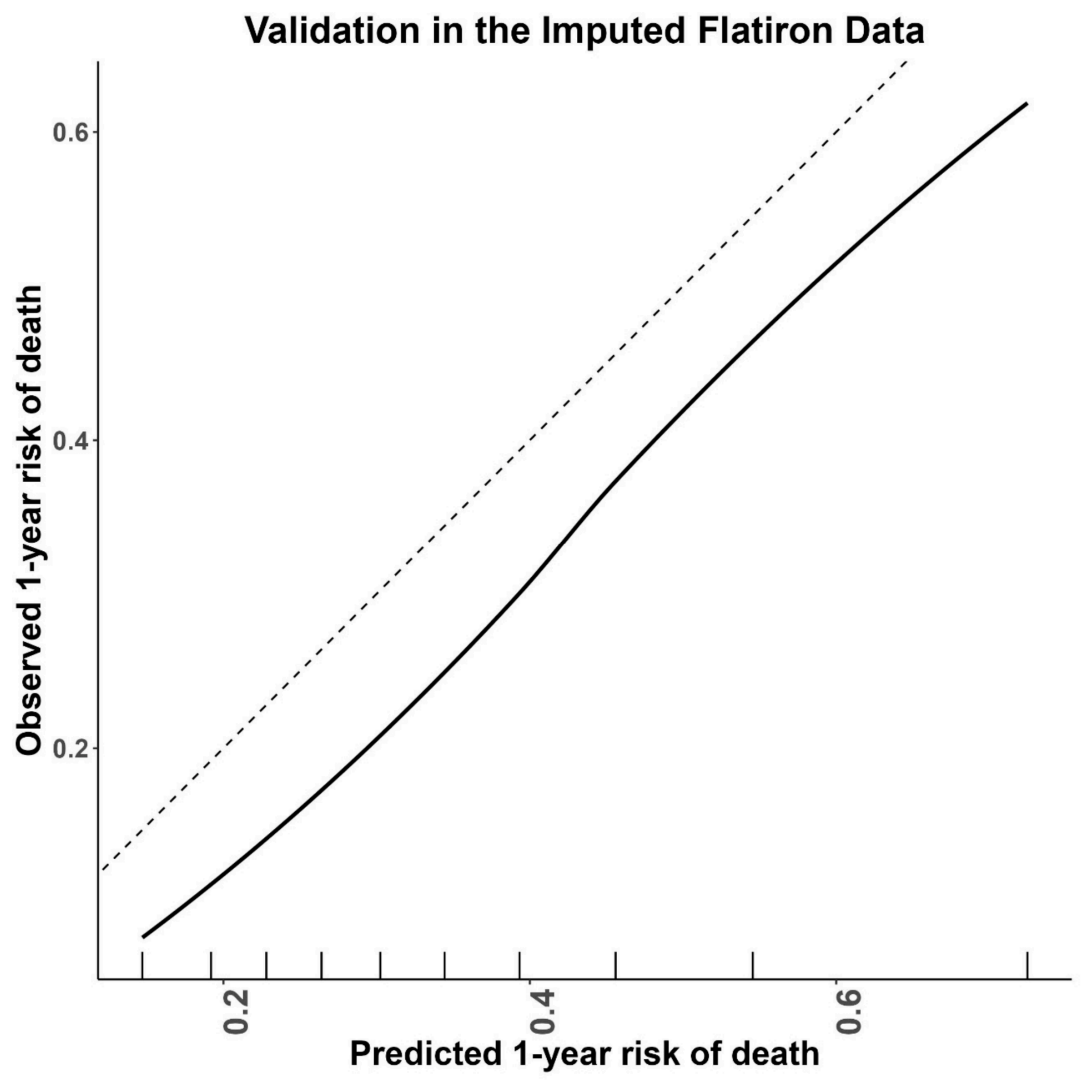
Calibration plot of the reduced ARCAD CRC nomogram in the real-world validation cohort. The solid black line represents the relationship between the predicted and observed 1-year risk of death. The dashed 45° line represents perfect calibration, where the predicted and observed 1-year risks are equal. A calibration curve below the 45° reference line indicates that the model overestimates the risk of death (or equivalently underestimates 1-year survival) in the validation cohort.

The reduced ARCAD CRC nomogram exhibited useful discrimination in our real-world validation cohort, with time-dependent AUROC at the 12-month time horizon of 0.741 (95% CI 0.729–0.753) (Figure 2). Uno’s C-index in the FRWC was 0.667 (95% CI 0.660–0.674). Calibration plots indicate that the reduced ARCAD CRC nomogram consistently underestimated the observed 1-year survival probability (Figure 3). The predicted 1-year survival rate by the reduced ARCAD nomogram and the actual observed 1-year survival rate in the overall Flatiron real-world cohort were 0.640 (95% CI 0.637–0.644) and 0.694 (95% CI 0.684–0.703), respectively.

The calibration slope in Figure 3 was 1.010 (95% CI 0.966–1.054), which is close to unity, indicating that the model retained an appropriate spread of predicted risks in the external validation cohort, with no evidence of substantial overfitting or underfitting. At the same time the calibration intercept was −0.160 (95% CI −0.189, −0.131), indicating systematic underestimation of 1-year survival in the FRWC. Consistent with this observation, the observed-to-expected event ratio was 0.852, indicating that fewer deaths occurred than predicted by the model.

## 4. Discussion

Novel, reliable prognostication can benefit both clinicians and patients in management decision-making. A nomogram is a tool used to predict the probability of a specific future event by emphasizing multiple contributing variables and has been extensively employed in cancer prognostication [9]. Multiple nomograms have been proposed for CRC; for example, the MSKCC CRC nomogram was designed to predict OS in early-stage colon cancer after curative resection [21]. The ARCAD CRC nomogram is one of the few available nomograms solely focused on survival prediction in stage 4 CRC. The ARCAD CRC was built on a cohort obtained from randomized phase 2/3 clinical trials. Therefore, to enhance generalizability and identify potential biases, external validation is warranted [11]. In 2020, a French group evaluated the performance of the ARCAD CRC nomogram in a small elderly cohort with 123 patients [22], with results presented in conference proceedings only. The ARCAD CRC nomogram showed mild calibration (Hosmer–Lemeshow calibration; *p* = 0.89) and moderate discrimination abilities (c-index, 0.66) [22]. To our knowledge, the current study is the first published study for the external validation of the ARCAD CRC nomogram in a large real-world cohort.

Clinical trial populations are often highly selected for several reasons, resulting from the use of strict inclusion and exclusion criteria and access to trial sites, interest of trial investigators, etc. [12,14]. This selectivity can introduce biases, thereby limiting the generalizability of findings from the trials. Our real-world validation cohort was older and had a poorer performance status than the original ARCAD clinical trial cohort did, as expected. Findings from our external validation indicate that the ARCAD CRC nomogram is still valuable in a real-world population, showing useful discrimination with time-dependent AUROC at the 12-month time horizon of 0.741. The AUROC, a time dependent measure, differs from the Uno’s C which is not time dependent. That the Uno’s C (0.667) is similar to the c-index for the reduced nomogram in the ARCAD cohort (0.67) is reassuringly consistent.

Notably, the ARCAD nomogram demonstrated acceptable discrimination, reflecting the model’s ability to stratify the low-risk and high-risk groups for death. It is important to distinguish between discrimination and calibration. While often a nomogram derived from clinical trial patients might overestimate survival due to the more selective nature of patients enrolled the ARCAD nomogram consistently underestimated survival (64.0% vs. 69.4%) in our real-world validation cohort. It is not possible to conclude the cause for the observed underestimation of survival by the ARCAD nomogram, but it may well be due to recent advances in treatment options for CRC, as the Flatiron real-world validation cohort was collected more recently (2013–2020) than patients enrolled in the ARCAD cohort (1997–2012) [11]. In any case the ARCAD nomogram performs well in terms of discrimination or ranking poorer versus better risk patients and a simple recalibration of the nomogram applied in real world settings may suffice to ensure its utility. The underestimation due to the aforementioned factor is likely one of the major contributing factors to the model prediction performance. Our findings indicate that the ARCAD CRC nomogram is a valuable decision-making tool for clinicians to predict 1-year survival in a real-world CRC cohort with stage 4 disease planned to receive systemic therapy. Clinicians may use the tool to distinguish between higher- and lower-risk patients. This tool can also potentially be utilized for prospective studies to assist investigators in selecting or stratifying patients or ensuring whether the risks are comparable between arms. Nonetheless, future work could also involve updating the ARCAD nomogram by using the same risk equation but recalibrating it to real-world patient populations.

One practical limitation of the ARCAD nomogram is its complexity and reliance on multiple continuous variables and molecular diagnostics, which can delay data acquisition and limit its utility as a real-time stratification tool. In our study, a reduced model using fewer variables achieved comparable prognostic performance, supporting its potential for more pragmatic use. By contrast, the Köhne Index employs a simpler, categorically defined set of routinely available variables (e.g., performance status, alkaline phosphatase, WBC count, and number of metastatic sites), making it more amenable to real-world stratification [23]. Similarly, a simplified model developed by Bachet et al. from studies across all lines of therapy in the ARCAD database utilized seven key patient and tumor characteristics common to all lines of treatment (performance status, hemoglobin, platelet count, WBC/absolute neutrophil count ratio, lactate dehydrogenase, alkaline phosphatase, and the number of metastatic sites) [24]. The substantial overlap with our models indicates that similar prognostic variables are applicable across a range of settings, treatments and timelines, and that our original ARCAD model is likely to remain relevant for risk stratifying. A head-to-head comparison of these prognostication tools would be valuable in future research.

The current studies are not without limitations. First, due to the unavailability of some variables, we generated a reduced ARCAD CRC nomogram model. This could have led to the loss of information and reduced predictive capacity. However, the contributing portion of these missed variables (prior chemotherapy, number of metastases, liver, lymph node, peritoneal metastases) ranks as the second to sixth smallest impact factors among the seventeen variables in the original full ARCAD CRC nomogram model [11]. Additionally, the C-index between the full model and the reduced model was nearly identical, with values of 0.67 and 0.68, respectively. Therefore, the generated reduced model in the current study retains similar performance to the full ARCAD CRC model despite using fewer variables and is potentially more feasible for real-world use. The FRWC used for validation only included patients who had received at least one dose of systemic therapy, consistent with the ARCAD population. Therefore the generalizability to participants not planned to receive therapy is unclear. Another limitation is imputation. *KRAS* and *BRAF* mutations were assumed to be mutually exclusive, however co-mutations, while rare, can occur. However, all of the independent variables were available for more than 65% of the patients, ranging from 0.0% to 32.2%. Mismatch repair and/or microsatellite instability status was not available; therefore, the impact of this factor and applicability of the model to subgroup of MSI-H tumors were not able to be assessed in this study [25,26,27]. Furthermore, recent therapeutic advances utilizing targeted approaches for *KRAS* G12C and *BRAF* V600E mutations were not incorporated into this real-world dataset, limiting the evaluation of their specific prognostic impact [28,29,30,31]. Future research would be valuable to update the model incorporating this information.

## 5. Conclusions

The ARCAD CRC nomogram was externally validated in a very large real-world cohort comprising 9710 patients. Our findings indicate that the ARCAD CRC nomogram is a useful decision-making tool for clinicians to predict 1-year survival in a real-world CRC cohort with stage 4 disease. The observed underestimation of survival by the ARCAD CRC nomogram in the real-world cohort is likely attributed to recent advances in CRC treatment options, such as greater incorporation of targeted therapy or immunotherapy into day-to-day practice, as the real-world validation cohort of the current study was collected more recently (2013–2020), in contrast to patients enrolled in the original ARCAD cohort (1997–2012).

## Figures and Tables

**Table 1:** Baseline characteristics of patients used for the reduced ARCAD CRC nomogram: real-world validation cohort and original ARCAD clinical trial cohort.

Characteristic	Flatiron Real-World External Validation Cohort * (*n* = 9710)	Original ARCAD Development Clinical trial Cohort * (*n* = 20,417) [11]	*p*-Value
**Age, years**			<0.001
Mean (SD)	62.8 (12.3)	61.3 (10.7)	
Median (IQR)	64.0 (54.0, 72.0)	62.0 (55.0, 69.0)	
**Sex**			<0.001
Male	5418 (56%)	12,580 (62%)	
Female	4292 (44%)	7837 (38%)	
**Body Mass Index**			<0.001
Mean (SD)	27.7 (6.4)	26.0 (4.8)	
Median (IQR)	26.9 (23.3, 31.0)	25.4 (22.7, 28.6)	
**ECOG-PS**			<0.001
0	4298 (44%)	10,940 (54%)	
1	3983 (41%)	8631 (42%)	
2+	1429 (15%)	846 (4.1%)	
***KRAS* Status**			<0.001
Mutant	4408 (45%)	8041 (39%)	
Wild-type	5302 (55%)	12,376 (61%)	
***BRAF* Status**			0.11
Mutant	768 (7.9%)	1727 (8.5%)	
Wild-type	8942 (92%)	18,690 (92%)	
**White blood cells, 10^9^/L**			0.7
Mean (SD)	8.5 (3.8)	8.5 (3.9)	
Median (IQR)	7.9 (6.3, 9.9)	7.8 (6.4, 9.7)	
**Hemoglobin, g/dL**			<0.001
Mean (SD)	11.8 (2.0)	12.4 (1.7)	
Median (IQR)	11.8 (10.3, 13.2)	12.4 (11.2, 13.6)	
**Platelets, 10^9^/L**			<0.001
Mean (SD)	327.3 (132.2)	334.0 (126.9)	
Median (IQR)	304.8 (236.0393.3)	309.0 (245.0, 397.0)	
**ANC, 10^9^/L**			<0.001
Mean (SD)	5.9 (3.1)	5.6 (2.6)	
Median (IQR)	5.3 (4.0, 7.0)	5.2 (4.0, 6.6)	
**Albumin, g/L**			<0.001
Mean (SD)	37.7 (5.3)	39.0 (5.4)	
Median (IQR)	38.0 (35.0, 41.0)	39.0 (36.0, 42.0)	
**Bilirubin, mg/dL**			<0.001
Mean (SD)	0.7 (1.2)	0.6 (0.9)	
Median (IQR)	0.5 (0.3, 0.7)	0.5 (0.3, 0.7)	

* Post-imputation data were utilized for both the Flatiron real-world external validation cohort and the original ARCAD development clinical trial cohort. Abbreviations: PS, performance status; ANC, absolute neutrophil count; IQR, interquartile range; SD, standard deviation.

**Table 2:** Cox models for 1-year survival prediction: reduced and full ARCAD CRC nomogram.

	Reduced Model				Full Model [REF]			
Variables	Coefficients	SE	HR (95% CI)	*p*-Value	Coefficients	SE	HR (95% CI)	*p*-Value
**Age, years**				<0.001				<0.001
	−0.0022	0.0017	†		−0.0012	0.0017	†	
	0.0101	0.0020			0.0078	0.0020		
**Sex**				0.005				0.01
Female	-	-	1.00 (Reference)		-	-	1.00 (Reference)	
Male	0.0516	0.0183	1.05 (1.02, 1.09)		0.0442	0.0186	1.05 (1.01, 1.09)	
**BMI, Kg/m^2^**	−0.0204	0.0045	†	<0.001	−0.0236	0.0046		<0.001
	0.0156	0.0054			0.0167	0.0055		
**ECOG-PS**				<0.001				<0.001
0	-	-	1.00 (Reference)		-	-	1.00 (Reference)	
1	0.3006	0.0180	1.35 (1.30, 1.40)		0.2663	0.0184	1.31 (1.25, 1.34)	
2+	0.6347	0.0393	1.89 (1.75, 2.04)		0.5471	0.0407	1.73 (1.58, 1.84)	
***KRAS***				<0.001				<0.001
Wild Type	-	-	1.00 (Reference)		-	-	1.00 (Reference)	
Mutant	0.3272	0.0177	1.39 (1.34, 1.44)		0.3000	0.0181	1.35 (1.30, 1.39)	
***BRAF***				<0.001				<0.001
Wild Type	-	-	1.00 (Reference)		-	-	1.00 (Reference)	
Mutant	0.7934	0.0293	2.21 (2.09, 2.34)		0.7922	0.0304	2.21 (2.09, 2.34)	
**WBC, 10^9^/L**	0.0066	0.0026	1.01 (1.00, 1.01)	0.011	0.0063	0.0027	1.01 (1.00, 1.01)	0.01
**Hemoglobin, g/dL**	−0.0379	0.0060	0.96 (0.95, 0.97)	<0.001	−0.0449	0.0063	0.96 (0.94, 0.97)	<0.001
**Platelets, 10^9^/L**				<0.001				<0.001
	0.0013	0.0002	†		0.0012	0.0002	†	
	−0.0014	0.0003			−0.0013	0.0003		
**Neutrophils, 10^9^/L**				<0.001				<0.001
	0.1168	0.0114	†		0.1129	0.0069	†	
	−0.0804	0.0116			−0.0767	0.0118		
**Albumin, g/L**				<0.001				<0.001
	−0.0494	0.0032	†		−0.0481	0.0032	†	
	0.0082	0.0035			0.0097	0.0036		
**Bilirubin, mg/dL**				<0.001				<0.001
	0.5039	0.0737	†		0.4842	0.0758	†	
	−0.5205	0.0808			−0.5016	0.0831		
**Prior chemotherapy**								<0.001
No					-	-	1.00 (Reference)	
Yes					0.1358	0.0237	1.15 (1.10, 1.20)	
**No. of met sites**								<0.001
0–1					-	-	1.00 (Reference)	
2+					0.1859	0.0224	1.20 (1.16, 1.26)	
**Liver metastasis**								<0.001
No					-	-	1.00 (Reference)	
Yes					0.1811	0.0240	1.20 (1.15, 1.26)	
**LN metastasis**								<0.001
No					-	-	1.00 (Reference)	
Yes					0.1375	0.0214	1.15 (1.10, 1.19)	
**Peritoneal metastasis**								<0.001
No					-	-	1.00 (Reference)	
Yes					0.1706	0.0248	1.19 (1.13, 1.22)	

† Single hazard ratio is not available due to nonlinear effect for these continuous variables. Abbreviations: BMI, Body Mass Index; CI, confidence interval; HR, hazard ratio; SE, standard error; LN, Lymph Node.

## Data Availability

H. Lee Moffitt Cancer Center holds responsibility for the accuracy of the clinical data and can provide data upon reasonable request.

## Supplementary Materials

The following supporting information can be downloaded at: https://www.mdpi.com/article/10.3390/cancers18183031/s1, Table S1: Baseline Characteristics of Patients Used for The Reduced ARCAD CRC Nomogram: Pre-imputation and post-imputation; Table S2: Missing counts of the variables in the FRWC before performing imputation; Figure S1: Flow diagram illustrating cohort selection; Figure S2: Complete-case sensitivity analysis (*n* = 4027).

## References

[1] Sung H., Ferlay J., Siegel R.L., Laversanne M., Soerjomataram I., Jemal A., Bray F. (2021). Global Cancer Statistics 2020: GLOBOCAN Estimates of Incidence and Mortality Worldwide for 36 Cancers in 185 Countries. CA Cancer J. Clin..

[2] Siegel R.L., Miller K.D., Fuchs H.E., Jemal A. (2022). Cancer statistics, 2022. CA Cancer J. Clin..

[3] Cancer of the Colon and Rectum—Cancer Stat Facts. SEER. https://seer.cancer.gov/statfacts/html/colorect.html.

[4] Yokota T., Ura T., Shibata N., Takahari D., Shitara K., Nomura M., Kondo C., Mizota A., Utsunomiya S., Muro K. (2011). *BRAF* mutation is a powerful prognostic factor in advanced and recurrent colorectal cancer. Br. J. Cancer.

[5] Yancik R., Wesley M.N., Ries L.A.G., Havlik R.J., Long S., Edwards B.K., Yates J.W. (1998). Comorbidity and age as predictors of risk for early mortality of male and female colon carcinoma patients. Cancer.

[6] Wichmann M.W., Müller C., Hornung H.M., Lau-Werner U., Schildberg F.W. (2001). Gender differences in long-term survival of patients with colorectal cancer. J. Br. Surg..

[7] Taieb J., Le Malicot K., Shi Q., Penault Lorca F., Bouché O., Tabernero J., Mini E., Goldberg R.M., Folprecht G., Luc Van Laethem J. (2017). Prognostic Value of *BRAF* and *KRAS* Mutations in MSI and MSS Stage III Colon Cancer. J. Natl. Cancer Inst..

[8] Stillwell A.P., Ho Y.H., Veitch C. (2011). Systematic Review of Prognostic Factors Related to Overall Survival in Patients with Stage IV Colorectal Cancer and Unresectable Metastases. World J. Surg..

[9] Iasonos A., Schrag D., Raj G.V., Panageas K.S. (2008). How To Build and Interpret a Nomogram for Cancer Prognosis. J. Clin. Oncol..

[10] Wang S., Liu Y., Shi Y., Guan J., Liu M., Wang W. (2021). Development and external validation of a nomogram predicting overall survival after curative resection of colon cancer. J. Int. Med. Res..

[11] Sjoquist K.M., Renfro L.A., Simes R.J., Tebbutt N.C., Clarke S., Seymour M.T., Adams R., Maughan T.S., Saltz L., Goldberg R.M. (2018). Personalizing Survival Predictions in Advanced Colorectal Cancer: The ARCAD Nomogram Project. JNCI J. Natl. Cancer Inst..

[12] Mansournia M.A., Higgins J.P.T., Sterne J.A.C., Hernán M.A. (2017). Biases in Randomized Trials. Epidemiology.

[13] Khozin S., Blumenthal G.M., Pazdur R. (2017). Real-world Data for Clinical Evidence Generation in Oncology. JNCI J. Natl. Cancer Inst..

[14] Abi Jaoude J., Kouzy R., Mainwaring W., Lin T.A., Miller A.B., Jethanandani A., Espinoza A.F., Pasalic D., Verma V., Vanderwalde N.A. (2020). Performance Status Restriction in Phase III Cancer Clinical Trials. J. Natl. Compr. Cancer Netw..

[15] Hamers P.A.H., Wensink G.E., Van Smeden M., Vink G.R., Smabers L.P., Lunenberg R.A., Laclé M.M., Koopman M., May A.M., Roodhart J.M.L. (2022). External Validation of the Colon Life Nomogram for Predicting 12-Week Mortality in Dutch Metastatic Colorectal Cancer Patients Treated with Trifluridine/Tipiracil in Daily Practice. Cancers.

[16] Stekhoven D.J., Bühlmann P. (2012). MissForest—Non-parametric missing value imputation for mixed-type data. Bioinformatics.

[17] Li Z.-N., Zhao L., Yu L.-F., Wei M.-J. (2020). *BRAF* and *KRAS* mutations in metastatic colorectal cancer: Future perspectives for personalized therapy. Gastroenterol. Rep..

[18] Núñez E., Steyerberg E.W., Núñez J. (2011). Regression Modeling Strategies. Rev. Esp. Cardiol. Engl. Ed..

[19] Ramspek C.L., Jager K.J., Dekker F.W., Zoccali C., Van Diepen M. (2021). External validation of prognostic models: What, why, how, when and where?. Clin. Kidney J..

[20] Grant S.W., Collins G.S., Nashef S.A.M. (2018). Statistical Primer: Developing and validating a risk prediction model†. Eur. J. Cardio-Thorac. Surg..

[21] Weiser M.R., Gönen M., Chou J.F., Kattan M.W., Schrag D. (2011). Predicting Survival After Curative Colectomy for Cancer: Individualizing Colon Cancer Staging. J. Clin. Oncol..

[22] De Rycke O., Tournigand C., Pujals A., Rouquette A., Blons H., Laurent M., Orvoen G., Rouquette P.B., Poisson J., Paillaud E. (2020). 512P External validity of the ARCAD nomogram in a real-life population of older patients with unresectable advanced colorectal cancer. Ann. Oncol..

[23] Köhne C.H., Cunningham D., Di Costanzo F., Glimelius B., Blijham G., Aranda E., Scheithauer W., Rougier P., Palmer M., Wils J. (2002). Clinical determinants of survival in patients with 5-fluorouracil- based treatment for metastatic colorectal cancer: Results of a multivariate analysis of 3825 patients. Ann. Oncol..

[24] Bachet J.-B., De Gramont A., Raeisi M., Rakez M., Goldberg R.M., Tebbutt N.C., Van Cutsem E., Haller D.G., Hecht J.R., Mayer R.J. (2025). Characteristics of Patients and Prognostic Factors Across Treatment Lines in Metastatic Colorectal Cancer: An Analysis From the Aide et Recherche en Cancérologie Digestive Database. J. Clin. Oncol..

[25] André T., Elez E., Lenz H.-J., Jensen L.H., Touchefeu Y., Van Cutsem E., Garcia-Carbonero R., Tougeron D., Mendez G.A., Schenker M. (2025). Nivolumab plus ipilimumab versus nivolumab in microsatellite instability-high metastatic colorectal cancer (CheckMate 8HW): A randomised, open-label, phase 3 trial. Lancet.

[26] André T., Shiu K.-K., Kim T.W., Jensen B.V., Jensen L.H., Punt C., Smith D., Garcia-Carbonero R., Benavides M., Gibbs P. (2020). Pembrolizumab in Microsatellite-Instability–High Advanced Colorectal Cancer. N. Engl. J. Med..

[27] Lenz H.-J., Lonardi S., Elez E., Van Cutsem E., Jensen L.H., Bennouna J., Mendez G., Schenker M., De La Fouchardiere C., Limon M.L. (2024). Nivolumab (NIVO) plus ipilimumab (IPI) vs chemotherapy (chemo) as first-line (1L) treatment for microsatellite instability-high/mismatch repair-deficient (MSI-H/dMMR) metastatic colorectal cancer (mCRC): Expanded efficacy analysis from CheckMate 8HW. J. Clin. Oncol..

[28] Elez E., Yoshino T., Shen L., Lonardi S., Van Cutsem E., Eng C., Kim T.W., Wasan H.S., Desai J., Ciardiello F. (2025). Encorafenib, Cetuximab, and mFOLFOX6 in *BRAF*-Mutated Colorectal Cancer. N. Engl. J. Med..

[29] Fakih M.G., Salvatore L., Esaki T., Modest D.P., Lopez-Bravo D.P., Taieb J., Karamouzis M.V., Ruiz-Garcia E., Kim T.-W., Kuboki Y. (2023). Sotorasib plus Panitumumab in Refractory Colorectal Cancer with Mutated *KRAS* G12C. N. Engl. J. Med..

[30] Kopetz S., Tabernero J., Lonardi S., Shen L., Wasan H.S., Yoshino T., Van Cutsem E., Kim T.W., Eng C., Ciardiello F. (2026). BREAKWATER: Progression-free and overall survival analyses of first-line (1L) encorafenib + cetuximab (EC) + FOLFIRI in BRAF V600E-mutant metastatic colorectal cancer (mCRC). J. Clin. Oncol..

[31] Yaeger R., Weiss J., Pelster M.S., Spira A.I., Barve M., Ou S.-H.I., Leal T.A., Bekaii-Saab T.S., Paweletz C.P., Heavey G.A. (2023). Adagrasib with or without Cetuximab in Colorectal Cancer with Mutated *KRAS* G12C. N. Engl. J. Med..

