# Supplementary material for: External Validation of the ARCAD Nomogram in a Real-World Cohort with Stage 4 Colorectal Cancer
Source: Cancers (Basel). 2026 Sep 18;18(18):3031. doi: 10.3390/cancers18183031 (PMC13605465; doi:10.3390/cancers18183031)
Supplement: Supplementary file 1 [file cancers-18-03031-s001.zip › cancers-4504499-supplementary.pdf]

cancers

MDPI

Supplementary Materials: External Validation of the ARCAD Nomogram in a Real-World Cohort with Stage 4 Colorectal Cancer

James Yu, Jiannong Li, Michael J. Schell, Pablo Gonzalez Ginestet, John Raymond Zalcberg, R John Simes, Ian Marschner, Richard D. Kim and Katrin Marie Sjoquist

Table S1. Baseline Characteristics of Patients Used for The Reduced ARCAD CRC Nomogram: Pre-imputation and post-imputation.

Characteristic	Pre-imputation No. (%)	Post-imputation No. (%)
**Age, yo**		
Mean (SD)	62.8 (12.3)	62.8 (12.3)
Median (IQR)	64.0 (54.0,72.0)	64.0 (54.0,72.0)
Number Missing	0 (0.0%)	0 (0.0%)
**Sex**		
Male	5,418 (56%)	5,418 (56%)
Female	4,292 (44%)	4,292 (44%)
Number Missing	0 (0.0%)	0 (0.0%)
**Body Mass Index**		
Mean (SD)	27.7 (6.5)	27.7 (6.4)
Median (IQR)	26.8 (23.2,31.2)	26.9 (23.3,31.0)
Number Missing	325 (3.3%)	0 (0.0%)
**ECOG-PS**		
0	4,298 (44%)	4,298 (44%)
1	3,983 (41%)	3,983 (41%)
2+	1,429 (15%)	1,429 (15%)
Number Missing	0 (0.0%)	0 (0.0%)
**KRAS Status**		
Mutant	3,281 (43%)	4,408 (45%)
Wild-type	4,302 (57%)	5,302 (55%)
Number Missing	2,127 (21.9%)	0 (0.0%)
**BRAF Status**		
Mutant	628 (9.5%)	768 (7.9%)
Wild-type	5,960 (90%)	8,942 (92%)
Number Missing	3,122 (32.2%)	0 (0.0%)
**White blood cells, 10^9/L**		
Mean (SD)	8.5 (3.9)	8.5 (3.8)
Median (IQR)	7.8 (6.2,10.0)	7.9 (6.3,9.9)
Number Missing	680 (7.0%)	0 (0.0%)
**Hemoglobin, g/dL**		
Mean (SD)	11.8 (2.0)	11.8 (2.0)
Median (IQR)	11.8 (10.3,13.2)	11.8 (10.3,13.2)
Number Missing	161 (1.7%)	0 (0.0%)
**Platelets, 10^9/L**		
Mean (SD)	326.8 (139.2)	327.3 (132.2)
Median (IQR)	300.5 (228.0,397.0)	304.8 (236.0,393.3)
Number Missing	1,362 (14.0%)	0 (0.0%)
**ANC, 10^9/L**		
Mean (SD)	5.9 (3.3)	5.9 (3.1)
Median (IQR)	5.2 (3.9,7.1)	5.3 (4.0,7.0)

Number Missing	2,438 (25.1%)	0 (0.0%)
**Albumin, g/L**		
Mean (SD)	37.7 (5.4)	37.7 (5.3)
Median (IQR)	38.0 (35.0,41.0)	38.0 (35.0,41.0)
Number Missing	577 (5.9%)	0 (0.0%)
**Bilirubin, mg/dL**		
Mean (SD)	0.7 (1.3)	0.7 (1.2)
Median (IQR)	0.5 (0.3,0.7)	0.5 (0.3,0.7)
Number Missing	697 (7.2%)	0 (0.0%)
Abbreviations: ANC, absolute neutrophil count; IQR, interquartile range; SD, standard deviation		

Table S2. Missing counts of the variables in the FRWC before performing imputation.

**Variable**	**N of missing**	**% of missing**
time	0	0
status	0	0
Age	0	0
gender	0	0
Performance_status	0	0
Hemoglobin	161	1.7
BMI	325	3.3
Albumin	577	5.9
WBC	680	7
Bilirubin	697	7.2
Platelets	1362	14
KRAS	2127	21.9
Neutrophils	2438	25.1
BRAF	3122	32.2

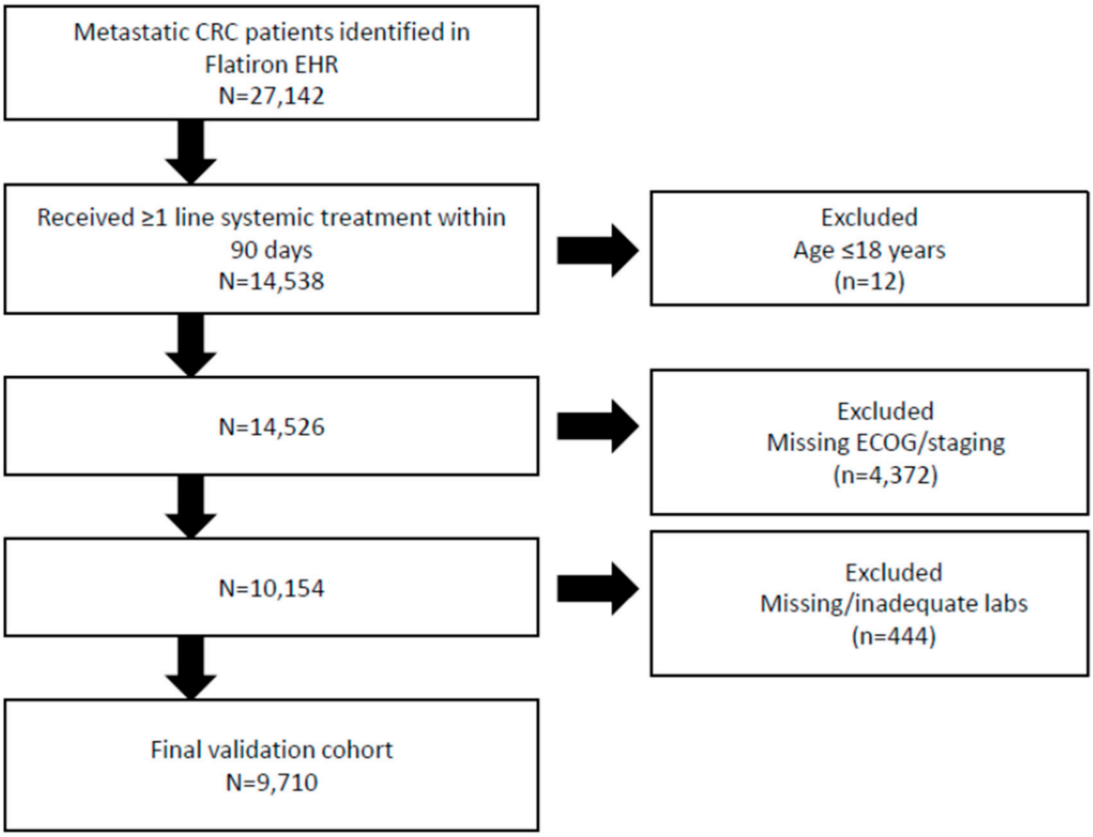

Figure S1. Flow diagram illustrating cohort selection.

(A)

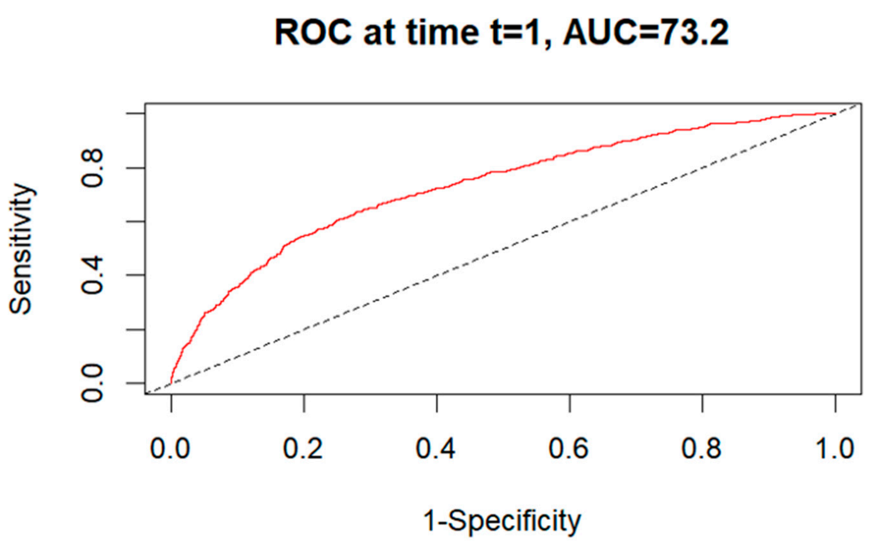

(B)

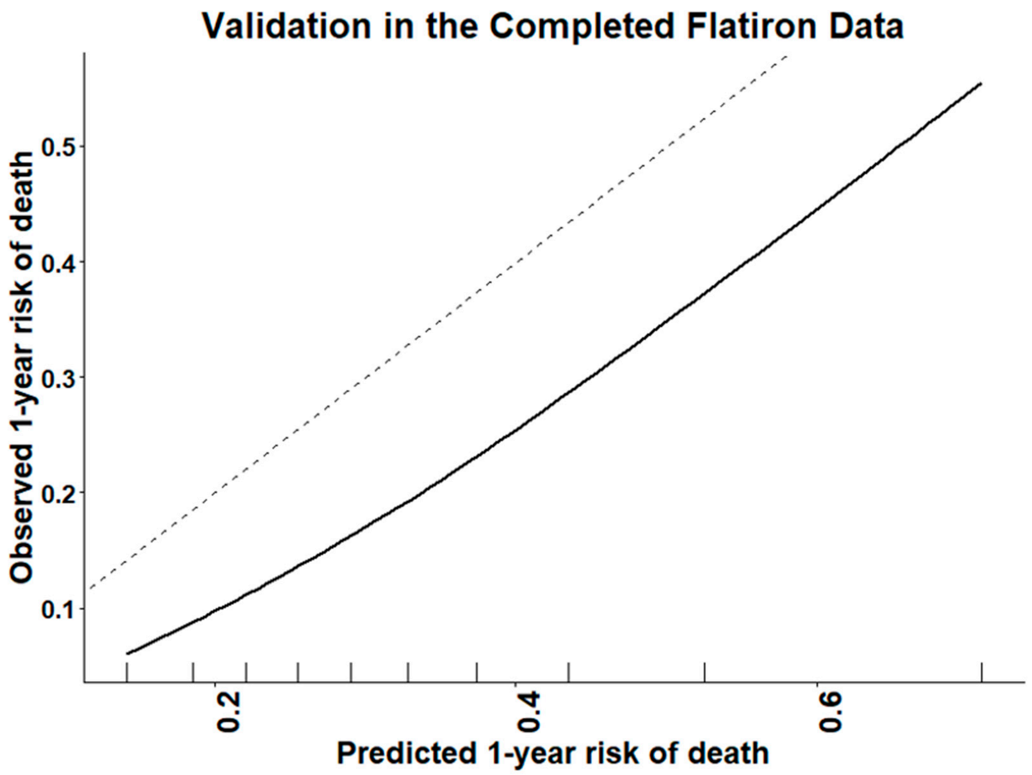

Figure S2. Complete-case sensitivity analysis (n = 4027). (A) Time-dependent Receiver Operating characteristic (ROC) curve for the reduced ARCAD CRC nomogram in the Flatiron real-world validation cohort, evaluated at 12 months to assess discrimination. The solid red curve represents the ROC curve of the reduced ARCAD model, with an AUC of 0.73.2. The dashed diagonal line represents the performance expected under random classification (AUC = 0.50). The greater the separation between the ROC curve and the diagonal reference line, the better the model's ability to discriminate between patients who did and did not experience death within 1 year. **(B)** Calibration Plot of the Reduced ARCAD CRC nomogram in the Real-World Validation Cohort. The solid black line represents the relationship between the predicted and observed 1-year risk of death. The dashed 45° line represents perfect calibration, where the predicted and observed 1-year risks are equal. A calibration curve below the 45° reference line indicates that the model overestimates the risk of death (or equivalently underestimates 1-year survival) in the validation cohort. s).
